# A Case of Hemorrhagic Ovarian Cyst Rupture Necessitating Surgical Intervention

**DOI:** 10.7759/cureus.29350

**Published:** 2022-09-20

**Authors:** Olivia Mantecon, Andrew George, Charlotte DeGeorge, Emily McCauley, Rohan Mangal, Thor S Stead, Bryna Peplinski, Latha Ganti

**Affiliations:** 1 Biology, Trinity Preparatory School, Winter Park, USA; 2 Pathology and Laboratory Medicine, Division of Biology and Medicine, Brown University, Providence, USA; 3 Emergency Medicine, HCA Florida Ocala Hospital, Ocala, USA; 4 Emergency Medicine, University of Central Florida College of Medicine, Orlando, USA; 5 Medicine, University of Miami Miller School of Medicine, Miami, USA; 6 Medicine, The Warren Alpert Medical School of Brown University, Providence, USA; 7 Obstetrics and Gynecology, Lakeland Regional Health Medical Center, Lakeland, USA; 8 Emergency Medicine, Envision Physician Services, Plantation, USA

**Keywords:** haemorrhagic ovarian cyst, corpus luteum cyst, ovarian cyst, hemoperitoneum, hemorrhagic ovarian cyst

## Abstract

Despite the relatively high incidence of ovarian cysts, particularly in premenopausal women, cyst rupture may on occasion present painfully and require surgical intervention to resolve. Particularly in the case of ruptured hemorrhagic ovarian cysts, resulting hemoperitoneum can create a risk of further adverse events including hypovolemic shock; proper identification and management of such cases are therefore critical. This case focuses on a 22-year-old female that presented to the emergency department (ED) with suprapubic pain in the lower left quadrant of the abdomen. Ultrasonography and computed tomography of the abdomen and pelvis revealed a ruptured hemorrhagic corpus luteum cyst of the left ovary and secondary hemoperitoneum. Patient treatment required laparoscopic left ovarian cyst wall removal, with the removal of hemoperitoneum.

## Introduction

Ovarian cysts are fluid-filled sacs that lie on the surface of or within the ovary. Roughly 20% of women develop at least one pelvic mass in their lifetime [1]. Postmenopausal women are less likely to develop ovarian cysts, thus premenopausal women are diagnosed with ovarian cysts at a much higher rate. Though several types of ovarian cysts exist, most are symptomless and often go undiagnosed. Functional cysts are the most common type of ovarian cysts; these are non-disease related, arise due to ovulation, and generally diminish over time without treatment. Though functional cysts can rupture without producing negative symptoms - the cyst fluid will dissipate and heal without treatment - 4% of women will be admitted to the hospital for ovarian cysts by the age of 65 [1]. Symptoms of ovarian cyst rupture include sudden severe abdominal pain, pain accompanied by vomiting, and lightheadedness or weakness.

Ovarian cysts can also be hemorrhagic. Hemorrhagic ovarian cyst (HOC) rupture can release blood and fluid into the surrounding abdomen and pelvis, presenting several risks to the patient including hemoperitoneum, less blood flow to vital organs, and sepsis. If the bleeding is severe, the patient may need to undergo laparoscopic surgery to control the bleeding or remove the cyst, as well as treatment to replace lost blood. We present a relatively rare case of a ruptured hemorrhagic corpus luteum cyst of the left ovary and consequent hemoperitoneum, treated with a laparoscopic left ovarian cyst wall removal.

## Case presentation

A 22-year-old female presented to the emergency department (ED) with a chief complaint of suprapubic pain in the lower left quadrant of the abdomen. The patient began to have pelvic cramping and feelings of weakness the night before and experienced 10 episodes of diarrhea and several episodes of vomiting throughout the night. She denied any vaginal bleeding, fevers, chills, chest pain, shortness of breath, headache, or urinary symptoms, had taken no medications for the pain, and had undergone no previous surgeries besides a tonsillectomy in second grade. Though the patient described her initial pain as resembling the pain she typically felt during menstruation, she had already menstruated previously in the month, and in this instance, her pain persisted instead of ultimately resolving. The patient had no related family medical history to contribute to a diagnosis.

Physical examination revealed vital signs within normal limits. Specifically, her temperature was 98.0°F, pulse 92 beats per minute, respirations 19 breaths per minute, blood pressure 145/73 mm Hg, and her oxygen saturation was 100% on room air. Examination of her heart and lungs was unremarkable, and the patient remained fully alert and oriented. Examination of the abdomen revealed suprapubic tenderness with voluntary guarding. Her laboratory analysis revealed leukocytosis, and anemia (Table 1).

**Table 1:** Patient’s laboratory analysis

Hematology	Range Reference	Result
White Blood Cell Count	4.0-10.5^3^/uL	14.5
Red Blood Cell Count	3.93-5.22 10^6^/uL	3.42
Hemoglobin	11.2-15.7 g/dL	11.0
Hematocrit	34.1-44.9%	31.4
Platelet Count	150-400 10^3^/uL	224
Chemistry	Range Reference	Result
Sodium	135-145 mmol/L	136
Potassium	3.5-5.3 mmol/L	3.5
Chloride	99-111 mmol/L	105
Carbon Dioxide	21-32 mmol/L	20 L
Anion Gap	3-12 mEq/L	14.5
Blood urea nitrogen	0.6-1.3 mg/dL	0.7
Estimated Glomerular Filtration Rate	>60	>60
Glucose	74-106 mg/dL	105
Lactic Acid	0.4-2.0 mmol/L	1.4
Calcium	8.4-10.2 mg/dL	8.8
Magnesium	1.8-2.4 mg/dL	1.6 L
Total Bilirubin	0.0-1.0 mg/dL	0.5
Direct Bilirubin	0.0-0.3 mg/dL	0.10
Indirect Bilirubin	0.2-0.8 mg/dL	0.4
Aspartate Aminotransferase	7-37 units/L	16
Alanine Aminotransferase	12-78 units/L	19
Total Alkaline Phosphatase	50-136/L	56
Total Protein	6.4-8.2 g/dL	6.5
Albumin	3.4-5.0 g/dL	4.0
Serum Human Choriogonadotropin Qualitative	NEGATIVE	NEGATIVE
Coagulation Studies	Range Reference	Result
Prothrombin Time	10.0-12.8 seconds	12.7 seconds
INR (International Normalized Ratio)	0.8-101	1.1
Partial Thromboplastin Time	25-38 seconds	25.5 seconds

**Figure 1:**
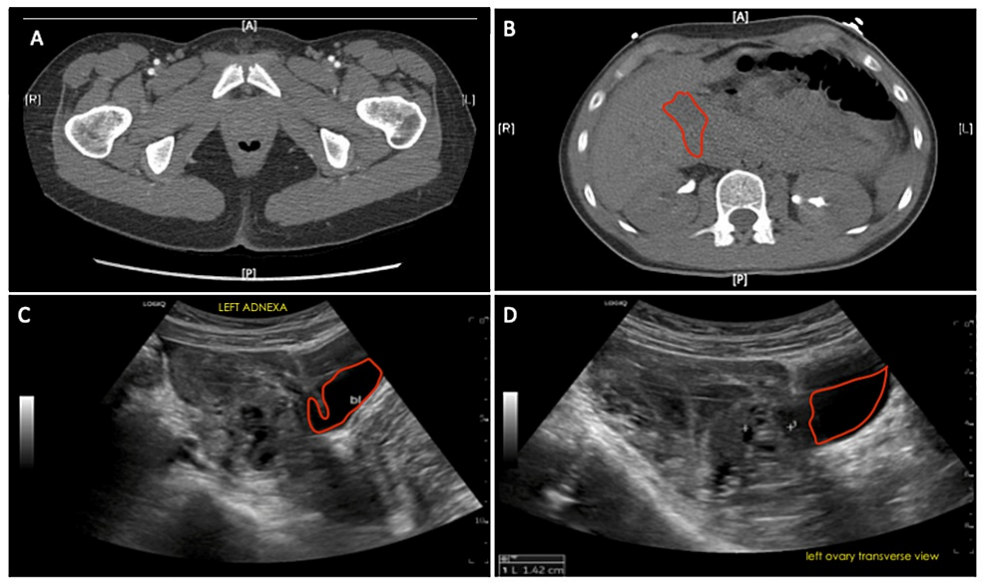
(A, B) CT of the pelvis and abdomen significant for an ill-defined hypodense structure is apparent in the left adnexa, measuring 4.4 x 3.1 cm with high-density fluid layering on top of the uterus and low to intermediate density abdominopelvic free fluid (red outline); hemorrhage is noted throughout the peritoneum and pelvis. (C, D) Non-obstetrical complete pelvic ultrasonography is significant for a complex septated cystic structure in the left ovary/adnexa and a large amount of free fluid (red outlines) and suspected blood in the pelvis, a portion of which appears complex.

Computed tomography scans of the abdomen and pelvis revealed an ill-defined hypodense left adnexal mass measuring 4.4 x 3.1 cm with high-density fluid layering on top of the uterus and low to intermediate density abdominopelvic free fluid (Figures 1A, 1B). This suggests the rupture of a HOC with hemoperitoneum. Transvaginal ultrasonography revealed a large amount of free fluid and suspected blood in the pelvis, likely representing hemorrhage, with a septated complex cystic structure in the left adnexa/ovary, providing concern for a hemorrhagic cyst (Figures 1C, 1D).

A gynecological consult following patient admission was used to guide further treatment. The patient was preoperatively diagnosed with a ruptured hemorrhagic corpus luteum cyst of the left ovary and hemoperitoneum. The probable presence of abdominal bleeding and the occurrence of leukocytosis presented a risk of sepsis for the patient. Based on the preoperative diagnosis and the patient’s worsening condition, a laparoscopic left ovarian cyst wall removal, with the removal of hemoperitoneum, was scheduled and performed. The post-operative diagnosis remained the same, with the addition of a diagnosis of pelvic adhesions. Post-surgical recovery was unremarkable, and the patient was later discharged home with appropriate instructions for continued care.

## Discussion

The most common type of ovarian cysts is functional cysts. These arise due to ovulation and generally resolve themselves within a few menstrual cycles. Complex cysts can develop due to other causes such as endometriosis and pelvic infections.

Among functional cysts, there are two types: follicular cysts and corpus luteum cysts. Follicular cysts occur upon the failure of the follicle to rupture and release the egg [2]. A successful egg release from the follicle leads to follicular production of estrogen and progesterone for conception. At this point, the follicle is called the corpus luteum. At times, the opening of the corpus luteum from where the egg was previously released can become blocked. If this occurs, the corpus luteum can accumulate fluid which causes it to develop into a corpus luteum cyst [3]. Other complex cysts are dermoid cysts, cystadenomas, and endometriomas. Dermoid cysts develop from the germ cells within the ovary; these rarely will be malignant [4]. Cystadenomas develop on the surface of the ovary and are usually filled with mucous material [5]. Endometriomas occur when cells similar to those within the uterus or endometrium grow outside the uterus. These cells can attach to the ovary and form a cyst.

Family history has been seen to play a role in the diagnosis of ovarian cysts and whether their function is abnormal. Caspi et al. conducted a case-control study in 2003 to determine the possible pathogenesis of ovarian dermoid cysts, assessing the relationship between significant first-degree family history and patient incidence [6]. Within the group of women with established diagnoses of dermoid ovarian cysts, 9.8% of women had at least one first-degree relative with a dermoid ovarian cyst, as compared with only 2% of women in the control group. This data suggests a genetic predisposition to dermoid ovarian cysts and provides a possibility of genetic disposition for other types of ovarian cysts. In this case, it is unknown whether the patient had first-degree relatives who had experienced functional ovarian cysts or ovarian cyst rupture.

Most ovarian cysts occur unnoticed and symptomless, however serious complications such as ovarian torsion and cystic rupture can happen. Dermoid cysts and cystadenomas can become large and cause the ovary to move out of position increasing the chances of the ovary becoming twisted; this can lead to a complete lack of blood flow to the ovary. Many ovarian cysts do not rupture, and amongst those that do, many ruptures without symptoms. However, at times, a ruptured ovarian cyst can lead to severe pain and hemoperitoneum, a possibly life-threatening condition. This occurs when cystic rupture leads to a release of fluid and blood into the abdomen or pelvis. Hemoperitoneum can result in hypovolemic shock, organ failure, organ damage, and even death.

When a female patient presents to the ED with symptoms similar to those of a ruptured ovarian cyst (sudden and severe abdominal pain, nausea and vomiting, weakness) the provider must determine whether the patient is pre or postmenopausal. If the patient is premenopausal, a serum beta hCG or urine pregnancy test is performed [1]. Medical imaging and abdominal and pelvic exams are then utilized as diagnostic tools. These allow the provider to determine whether the patient is experiencing a ruptured ovarian cyst or an alternate abdominal concern. Blood counts can be utilized to determine whether the patient is anemic due to acute bleeding or hemorrhage from the cyst; hemoglobin and hematocrit levels should be examined. If the provider or OB/GYN physician determines that the patient has a ruptured ovarian cyst, there are a few options. If the patient has mild symptoms and no bleeding in their abdomen, this condition can be mitigated with pain medication and monitoring of symptoms with few risks. However, severe cases with uncontrolled abdominal bleeding or hemorrhage can require surgery, as did the case described herein.

Several surgical options for ruptured HOCs exist based on the severity of bleeding [7]. However, the preferred method is through laparoscopy, due to its precision for abdominal and pelvic areas, especially in women. Further advantages when compared to open surgery include minimal scarring, shorter recovery times, and less painful recovery. When performing a laparoscopy, the surgeon will make a small incision around 1-1.5 cm near the umbilicus, through which a small tube with a camera is inserted; then CO_2_ is pumped through the tube to inflate the abdomen.

## Conclusions

In this case, the patient was diagnosed with a ruptured corpus luteum cyst with hemoperitoneum and treated laparoscopically for a left ovarian cyst wall removal, with the removal of hemoperitoneum. Although there is a relative infrequency of need for surgical intervention in ovarian cysts rupture, this case highlights the possible severity of HOCs. Consequently, it is important to consider all patients presenting to the ED with symptoms of ovarian cyst rupture for the possibility of hemoperitoneum. Proper identification and monitoring of such conditions are critical to preventing adverse outcomes.
